# Supplementary material for: The genome sequence of the Ruddy Streak moth, Tachystola acroxantha (Meyrick, 1885)
Source: Wellcome Open Res. 2024 May 20;9:276. [Version 1] doi: 10.12688/wellcomeopenres.21584.1 (PMC11862363; doi:10.12688/wellcomeopenres.21584.1)
Supplement: Peer review history

# Reviewer report: Reviewer response for version 1

**DOI:** 10.21956/wellcomeopenres.23856.r99838

**Published:** 2025-02-25

**Authors:** Carlos Lopez-Vaamonde

**Affiliations:** 1 INRAE Centre Val de Loire, Monnaie, France

The assembly quality value, k-mer completeness, BUSCO completeness score, and the percentage of the assembly assigned to chromosomes all indicate a high-quality assembly with significant coverage and completeness.

 Please find below a few minor comments for the authors to consider:

1. **Title**: Please include the order and family (Lepidoptera, Oecophoridae) after the species name.
2. **DNA Barcode & Metadata:** I strongly recommend depositing the DNA barcode and all associated metadata in the BOLD (Barcode of Life Data) system, including all relevant details such as the specimen's identifier. This species also appears to have two major mitochondrial lineages—BOLD:AAV3618 & BOLD:AAV3618—occurring in Australia and the UK. It would be valuable to verify whether the barcode of the voucher clusters within one of these mitochondrial lineages.

Are sufficient details of methods and materials provided to allow replication by others?

Yes

Is the rationale for creating the dataset(s) clearly described?

Yes

Are the datasets clearly presented in a useable and accessible format?

Yes

Are the protocols appropriate and is the work technically sound?

Yes

Reviewer Expertise:

Molecular systematics of Lepidoptera

I confirm that I have read this submission and believe that I have an appropriate level of expertise to confirm that it is of an acceptable scientific standard.

# Reviewer report: Reviewer response for version 1

**DOI:** 10.21956/wellcomeopenres.23856.r104522

**Published:** 2024-11-13

**Authors:** Giobbe Forni

**Affiliations:** 1 University of Bologna, Bologna, Italy

The manuscript by Crowley *et al*. reports the genome assembly of *Tachystola acroxantha*, the first genome resource available for this genus of moths. Leveraging PacBio HiFi long-read sequencing and Hi-C chromosome conformation capture, the authors have achieved a high-quality, chromosome-level assembly. The methodologies are sound and reflect current best practices in genome assembly, resulting in strong summary statistics that highlight the genome’s quality. This resource will be highly valuable for research into the geographic spread of alien species and the genetic mechanisms underlying local adaptation. Overall, this genome note meets high standards and I have no major comments or concerns.

Are sufficient details of methods and materials provided to allow replication by others?

Yes

Is the rationale for creating the dataset(s) clearly described?

Yes

Are the datasets clearly presented in a useable and accessible format?

Yes

Are the protocols appropriate and is the work technically sound?

Yes

Reviewer Expertise:

Evolution; Phylogenetics

I confirm that I have read this submission and believe that I have an appropriate level of expertise to confirm that it is of an acceptable scientific standard.
